# The Long-Term Outcomes of Pulmonary Hypertension in Systemic Lupus Erythematosus (SLE-PAH) Vary Among Different Autoantibody-Based Clusters: An Observational, Longitudinal Study Conducted at a Single Centre in India

**DOI:** 10.31138/mjr.060425.acl

**Published:** 2025-08-26

**Authors:** Ritasman Baisya, Yerram Keerthi Vardhan, Murthy GSR, Yerram Vivek Vardhan, Liza Rajasekhar

**Affiliations:** 1 Rheumatology & Clinical Immunology, Nizam’s Institute of Medical Sciences, Hyderabad, India;; 2 Indian Statistical Institute, Hyderabad, India;; 3 Statistics, Oklahoma State University, Stillwater, Oklahoma, United States of America

**Keywords:** Systemic Lupus Erythematosus, pulmonary hypertension, cluster analysis, autoantibodies, prognosis

## Abstract

**Background:**

Pulmonary arterial hypertension (PAH) is often overlooked in systemic lupus erythematosus (SLE) patients. However, the association between SLE and PAH is gaining attention due to unique mechanisms and treatment responses.

**Objectives:**

This study aims to categorise SLE-PAH patients by autoantibody profiles and evaluate long-term outcomes, including mortality and changes in PAH over 12 months.

**Method:**

A hospital-based investigation included SLE patients diagnosed with PAH. We analysed mortality, PAH resolution, increases in anti-PAH therapy, and follow-up right ventricular systolic pressure (RVSP) over 12 months. K-means cluster analysis was used for clustering, with regression analyses predicting mortality.

**Result:**

Analysing 111 SLE PAH patients revealed three clusters: Cluster 1 (CL1)—Sm-RNP positive (n=48), Cluster 2 (CL2)—no specific autoantibodies (n=36), and Cluster 3 (CL3)—multiple autoantibody positivity (n=27) (Smith, Sm-RNP, nucleosome, histone). Nephritis was significant in CL3 with multiple antibody positivity compared to CL1 and CL2 (p=0.01). CL1 showed a higher baseline mean RVSP (64.5 ± 19.1 (CL1) vs 57.5 ± 12.6 (CL2) vs 50.9 ± 12, p=0.002) and more frequent use of dual anti-PAH therapy (p=0.000). CL2, without any specific antibody, had an earlier onset of PAH (p=0.04). Significant improvement in PAH after withdrawing anti-PAH medication was noted in Cluster 3 (p=0.00). Regression analysis showed that aggressive anti-PAH therapy lowers PAH-related mortality (p=0.01), while nephritis (p=0.012) and positive dsDNA (p=0.009) correlate with higher mortality.

**Conclusion:**

This study is the first to analyse SLE-PAH through cluster-based methods. Sm-RNP antibodies indicate more severe disease needing dual therapy, while multiple positive antibodies suggest a better prognosis, with most patients achieving PAH resolution.

## INTRODUCTION

Pulmonary arterial hypertension (PAH) is a significant yet frequently overlooked complication of systemic lupus erythematosus (SLE). There is ongoing debate regarding the inflammatory characteristics of PAH in SLE, with cluster analysis suggesting that PAH may present in two phenotypes: vasculitic (inflammatory) and vasculopathic.^1^ A recent study indicated that many SLE-PAH patients experienced regression of PAH following immunosuppressive therapy.^2^ However, limited data on PAH among Indian lupus patients emphasise the necessity for further research in India, especially regarding predictors and long-term outcomes. We have previously identified three distinct clusters of SLE-PAH patients based on their autoantibodies.^3^ Although some multicentric studies have investigated long-term prognoses and risk factors, none have compared outcomes across these different clusters.^4,5^ This study intends to explore the long-term outcomes in these distinct SLE-PAH patient clusters.

## OBJECTIVES

The primary objective of this study was to categorise patients with SLE-PAH into various clusters based on their autoantibody profiles. We aimed to evaluate long-term outcomes, including mortality and changes in PAH (improvement or worsening) over a 12-month period. Additionally, we conducted a regression analysis to identify predictors of mortality related to SLE-PAH in our study population.

## METHOD

### Study design

This Institution-based longitudinal study was conducted over two years at a tertiary care hospital in South India.

### Participants

Patients with SLE diagnosed PAH according to defined criteria were included as cases. Data were collected over a 12-month period, and each patient was followed up for an additional 12-month period. The study received approval from the institutional ethics committee (EC 48th NIMS-ESGS/1024/2020), and written informed consent was obtained from all patients, including separate consent forms for minors, before participation.

### Inclusion Criteria

The following criteria were established for inclusion in the study:

- SLE patients, both juvenile and adult, fulfilling the “SLICC 2012” (SLE International Collaboration Clinics [SLICC] classification criteria)^6^ or ACR–EULAR 2019 (American College of Rheumatology - European League Against Rheumatism) classification criteria^7^ evaluated as outpatient or admitted as inpatient with PAH as a cardiopulmonary complication were included as study participants.
- PAH was defined solely in the context of clinical signs, which include dyspnoea not attributed to other causes, a loud P2 (pulmonary component of the second heart sound), and transthoracic echocardiography showing a right ventricular systolic pressure (RVSP) greater than 40 mm Hg or a tricuspid regurgitate velocity (TRV) exceeding 3.4 m/s. For a few patients, a right heart catheterisation was performed with a cutoff mean pulmonary arterial pressure (PAP) greater than 20 mm Hg (most of the patients were ill and did not consent to the invasive procedure).

### Exclusion criteria

The study excluded patients with previously diagnosed cardiac diseases, including rheumatic heart disease (RHD) and congenital heart disease, before the onset of lupus, as well as pregnant and lactating patients.

### Study variables

Demographic variables such as age, sex, duration of the disease, and duration of PAH were recorded. The clinical history included a detailed account of various organ involvement and the Systemic Lupus Erythematosus Disease Activity Index (SLEDAI). A comprehensive history of steroid use, immunosuppressive medications (including cyclophosphamide), and PAH treatments (such as phosphodiesterase-5 inhibitors like sildenafil or tadalafil, and endothelin receptor antagonists like ambrisentan) was also collected. Patients with an SLEDAI score of 6 or higher were classified as having high disease activity. In contrast, those with a SLEDAI score < 6 and without evidence of haemolytic anaemia, thrombocytopenia, or significant organ involvement were considered to have low disease activity.

### Disease outcome

We analysed mortality, PAH reversion (normalisation of RVSP with clinical improvement), increments in anti-PAH therapy, and RVSP measurements at the end of the 12-month follow-up period.

### Antibody assay

ANA immunoblot (EUROLINE test kit) provided in vitro determination of IgG class’s human autoantibodies in serum against 12 different antigens: Sm-RNP, Smith, SS-A, SS-B, dsDNA, nucleosome, histone, a ribosomal P protein, PCNA, PM-Scl, Jo-cyt1, and AMA-M2. Euro immune recommends interpreting results based on signal intensity. Signal intensity of more than 25 (>25) in the EURO-Line Scan Flatbed scanner generated a medium to strong band and was considered a positive result for the particular antibody. An anticardiolipin (ACL) antibody test (both immunoglobulin G [IgG] and immunoglobulin M [IgM]) was performed on all patients using the enzyme-linked immunosorbent assay (ELISA) technique. ACL IgG > 11 GPL and IgM > 15 MPL were considered positive. Serum complement level was assessed using immunoturbidimetry, and C3 < 90 mg/dL and C4 < 10 mg/dL were considered hypocomplementemia.

### Statistical analysis

K-means cluster analysis (non-hierarchical clustering or Quick Cluster; SPSS version 10 software; SPSS) was used to identify clustering in SLE-PAH patients based on autoantibody. ANOVA and chi-square tests were performed for quantitative and qualitative variables. Logistic regression analysis was performed to predict variables of SLE-PAH outcome. A p-value less than 0.05 was considered significant.

## RESULTS

### Demography and clinical characteristics

A total of 111 SLE PAH patients were included in the study, with a female-to-male ratio of 106:5 and a mean age of 28.9 years (± 9.2). The median duration of the disease was 36 months (IQR 36), and the time gap between the diagnosis of SLE and PAH was 12 months (IQR 36). Twenty patients experienced Raynaud’s phenomenon, and one patient had digital gangrene. The most commonly involved domains were musculoskeletal issues, followed by mucocutaneous manifestations, cytopenia, and nephritis. Pulmonary involvement was observed in various forms—such as pneumonitis, interstitial lung disease, and nodules—in 22 patients, one of whom presented with diffuse alveolar haemorrhage. A detailed description of the clinical phenotype is presented in **Table 1**.

**Table 1.** Clinical profile of SLE-PAH.

**Variables (N= 111)**	**Frequency**	**Percentage**
Mean age (years)	28.9 ± 9.2	
Disease duration (Median, months)	36 (36)	
Gap of disease and PAH (Median, months)	12 (12)	
Mean RVSP at baseline (mm Hg)	62.18 ± 17	
Mucocutaneous	88	79.3
Musculoskeletal	91	82
Raynaud Phenomenon	20	18
Nephritis	60	54
Vasculitis	18	16.2
Serositis	54	48.6
Neuropsychiatric lupus (NPSLE)	8	7.2
Mononeuritis multiplex	5	4.5
Cytopenia	68	61.3
Pulmonary involvement	22	19.8

### Outcome of PAH

The mean RVSP at the beginning of the study was 62.18 ± 17 mm Hg. After a 2-year follow-up, 13 patients succumbed, with nine deaths attributed to PAH. 47.2% of patients showed an improvement in RVSP at the end of the two years. PAH worsened in 22 patients. Cyclophosphamide was used by 78 patients, but it was not the primary treatment for PAH in all cases. Dual anti-PAH therapy, including an endothelin antagonist and PDE5 inhibitor, was administered to 42 patients. A detailed description of the outcome is depicted in **Table 2**.

**Table 2.** Two years’ outcome in SLE-PAH.

**Variables**	**Frequency**	**Percentage**
Reversal of PAH	51	47.2
Death	13	11.7
Death attributed to PAH with disease activity	9	8.1
Increase in PAH	21	18.9
Dual anti-PAH (endothelin anta + PDE5 inhibitor)	42	37.8
Cyclophosphamide	78	70.3

### Cluster analysis with outcome

Three distinct clusters were generated using K means cluster analysis – Cluster 1 had predominant Sm-RNP positivity with the maximum number of patients (n= 48), followed by Cluster 2 (no definite auto-antibody, n= 36) and Cluster 3 having multiple autoantibody positivity (Smith, Sm-RNP, Nucleosome and Histone). Anti-cardiolipin antibody and Ribosomal P protein failed to form any cluster. Over a follow-up period of 12 months, 13 patients succumbed, with the highest number in cluster 1 (clinically not significant). Cluster 2, with no definite antibodies, presented with PAH earlier than the other two clusters (p- 0.01). Improvement of PAH (RVSP < 40 mm Hg) with the subsequent withdrawal of anti-PAH medication was statistically significant in cluster 3 (p- 0.00), while higher baseline mean RVSP (p=0.04) and use of dual anti-PAH therapy (PDE5 inhibitor and endothelin antagonist) (p- 0.000) were statistically significant in cluster 1. The comparison of different clusters is depicted in **Table 3.**

**Table 3.** Comparison of clinical characters and outcomes in different clusters of SLE-PAH.

**Variables**	**Cluster 1**	**Cluster 2**	**Cluster 3**	**p-value**
Auto-antibodies	Sm-RNP alone	No antibodies	Nucleosome, Histone, Smith, Sm-RNP	-
Number of patients	48	36	27	-
Raynaud phenomenon	11	4	5	0.38
Nephritis	20/48	19/36	21/27	**0.01**
Serositis	25/48	14/36	15/27	0.35
Mean SLE duration (m)	41.02 ± 28.85	45.34 ±32.489	53.33 ± 29.02	0.26
Interval between SLE and PAH onset (m)	15.81± 22.87	5.26 ±12.07	10.30 ± 17.48	**0.04**
Mean RVSP (mm Hg) at baseline	64.52±19.17	57.51 ±12.69	50.96 ± 12.01	**0.002**
Mean RVSP at follow-up (patients not having a resolution of PAH)	65.2±24.8	61.4±17.8	56.33±21.9	-
Death	5/43 (11.6)	6/33 (18.2)	2/27 (7.4)	0.44
Death attributed to PAH	4/42 (9.5)	5/33 (15.2)	0/27 (0)	-
Resolution of PAH (Clinical asymptomatic and RVSP < 40 mm Hg on 2D echo)	14/40 (35)	15/31 (48.4)	22/27 (81.5)	**0.00083**
Worsening of PAH	9/39 (23.1)	9/31 (29)	3/27 (11.1)	0.25
Dual anti-PAH therapy	24/38 (63.2)	11/31 (35.5)	7/27 (25.9)	**0.006**
Cyclophosphamide	32/41 (78)	24/32 (75)	22/27 (81.5)	

The regression analysis revealed that certain factors significantly impact the likelihood of death in patients with SLE-PAH. Specifically, an increase in anti-PAH therapy is associated with a statistically significant reduction in the risk of disease-related death, indicated by a positive coefficient of 3.67 and a p-value of 0.010. This suggests that following an increased therapy regimen substantially lowers the mortality risk for SLE patients. On the other hand, the presence of nephritis and anti-dsDNA antibodies is linked to a higher likelihood of SLE-PAH-related death. Nephritis has a negative coefficient of −2.76 with a p-value of 0.012, while anti-dsDNA antibodies have a positive coefficient of 3.10 and a p-value of 0.009, indicating significant associations. (**Table 4**)

**Table 4.** Regression analysis indicated that various factors significantly influence the likelihood of death in patients with SLE-PAH.

**Coefficients Term**	**Coefficient**	**SE Coefficient**	**Z-Value**	**P-Value**
Constant	−0.18	2.21	−0.08	0.933
**SLE duration in month**	**−0.059**	**0. 289**	**−2.06**	**0.04**
SLE diagnosis -PAH onset gap month	−0.039	0. 549	−0.72	0.473
Initial RVSP > or equal to 40 mm Hg	−0.023	0. 330	−0.7	0.482
Latest RVSP	0.002	0. 232	0.07	0.941
Use of Cyclophosphamide	−1.63	1.06	−1.54	0.124
**Increment of therapy**	**3.67**	**1.42**	**2.59**	**0.01**
**Nephritis**	**−2.76**	**1.09**	**−2.52**	**0.012**
Cytopenia	0.465	911	0.51	0.61
**dsDNA**	**3.1**	**1.18**	**2.63**	**0.009**
Anti-cardiolipin antibody	1.405	969	1.45	0.147

## DISCUSSION

Our current study identified three distinct clusters based on autoantibody associations. Cluster 1, characterised by a predominant presence of anti-Sm-RNP antibodies, demonstrated significantly high baseline PAH and required dual PAH-targeted therapy. Conversely, Cluster 3, which exhibited multiple autoantibody positivity, showed an improvement in PAH over time, leading to the withdrawal of treatment.

We conducted a brief literature review using the MeSH terms (systemic lupus erythematosus, pulmonary hypertension, autoantibodies, cluster analysis, prognosis) in databases such as MED-LINE (PubMed), Web of Science, Embase, Scopus, and the Directory of Open Access Journals for the last ten years (2015 to April 2025). We found limited studies on SLE-PAH where cluster analysis was performed to identify the PAH phenotype and prognosis.

In a study conducted by Dai et al.^8^ focusing on 163 SLE-PAH patients, two distinct clusters were identified using consensus clustering analysis. Cluster 2, which had fewer patients, exhibited high disease activity, including nephritis and cardiac involvement, along with elevated mean SLEDAI scores. This cluster also showed a lower frequency of pulmonary-targeted treatment (PTD). Notably, they did not find any significant association between antibodies and SLEPAH. They highlighted older age at diagnosis, anti-dsDNA levels, neuropsychiatric lupus, and platelet distribution width as prognostic indicators of SLE-PAH-related mortality. In our study, we utilised autoanti-body-based clustering. We found that Cluster 3, with multiple antibody positivity, had higher rates of nephritis and elevated SLEDAI scores, along with an early withdrawal from PTD. Our predictive model indicated that nephritis and dsDNA levels are predictors of SLE-PAH mortality.

Huang et al.^9^ included 111 RHC-confirmed SLE-PAH patients with an onset age of 34.6 ± 8.6 years old and an average SLE duration of 5 years. Six independent variables were discovered: baseline SLE duration, interstitial lung disease, without acute rash, anti-SSA antibody), SLEDAI≤9, ESR≤20 mm/h, and uric acid > 357 μmol/L) to be associated with PAH in SLE patients. In the present study, a significant number of patients exhibited pulmonary involvement. However, this factor was not a reliable predictor of disease outcomes.

Our study identified Cluster 1, which is linked to a high occurrence of Sm-RNP antibodies. This cluster displayed more severe baseline PAH and a greater need for dual therapy. A study^10^ found that patients with SLEPAH who were positive for anti-U1 RNP antibodies had lower survival rates. Specifically, survival rates in the anti-U1 RNP-positive group were 94.4% after one year, 81.1% at three years, and 65.6% at five years. In contrast, the anti-U1 RNP-negative group’s survival rates were 95.0% at one year, 90.0% at three years, and 79.4% at five years, resulting in a hazard ratio of 1.46 (95% confidence interval 0.62–3.32, P = 0.3995). A recently published systematic review encompassed 21 studies and analysed various risk factors that were strongly associated with the development of SLE-PAH, including peak tricuspid regurgitation velocity on echocardiography >2.8 m/s, pericardial effusion, DLCO <70% predicted, scleroderma pattern on nailfold capillaroscopy, brain natriuretic peptide >50 ng/l, and N-terminal pro–brain natriuretic peptide >300 ng/l. However, they did not consider anti-RNP antibodies as a potential risk factor following multiple rounds of Delphi panel discussions.^11^

Baseline and follow-up data were gathered from a multicentre study known as the Cstar PAH cohort.^4^ The primary endpoint of the study was mortality. During a follow-up period of up to 5 years, there were 42 recorded deaths, accounting for 14.9% of the cohort. The survival rates were 92.1% at 1 year, 84.8% at 3 years, and 72.9% at 5 years. A multivariate Cox regression analysis, adjusted for sex and age, found a cardiac index of less than 2.5 L/min/m^2^ to be an independent predictor of mortality, with a hazard ratio (HR) of 2.62 (p = 0.006). However, it is important to highlight that right heart catheterisation was not conducted on most patients in the study.

A recent study conducted by Yaqi Zhao et al. involved 205 SLE-PAH patients and revealed five distinct clusters based on their clinical and serological traits. The findings indicated that the cluster group experienced a higher incidence of lupus nephritis (LN) among SLE-PAH patients, highlighting that dsDNA and the Neutrophil-lymphocyte ratio predicted the relationship between nephritis and PAH.^12^ Our research also demonstrated that patients with multiple positive antibodies had a significantly increased risk of nephritis. This leads us to hypothesise that not all PAH in SLE is attributable to damage; instead, it may correlate with disease activity that responds positively to immunosuppressive therapy. Another study^2^ observed that over one-third of SLE-PAH patients achieved complete resolution of PAH (based on right heart catheter assessment) following immunosuppressive treatment, alongside reduced mortality risk and improved NYHA functional class scores, further supporting the notion that inflammation could significantly influence SLEPAH.

Research has shown that immunosuppressive therapy can predict short-term survival in patients with connective tissue disease-related pulmonary arterial hypertension (CTD-PAH).^13^ An earlier open-label observational study at the same centre indicated that treatment with immunosuppressants might significantly improve PAH.^14^ However, our study did not find evidence that immunosuppressive therapy predicts mortality. Additionally, no significant difference was observed in the use of immunosuppressive treatment among the clusters.

This study has several limitations. First, not all patients underwent right heart catheterisation; therefore, PAH diagnosis and reversal were based on clinical and echocardiographic parameters. Additionally, serum biomarkers, such as N-terminal BNP, were not included as measures of disease outcomes for which stratification based on risk factors would not be possible in this study. However, this is a potential area of interest in future work. The follow-up period was also relatively short. Lastly, a quantitative comparison of antibody levels was not conducted in this study.

## CONCLUSION

This research is the first to explore the long-term follow-up of SLE-PAH through cluster analysis, focusing on autoantibodies. Rheumatologists must be aware of the potential occurrence of PAH in patients with SLE.^15^ The detection of Sm-RNP antibodies indicates a more severe disease form necessitating dual PAH therapy. Notably, the presence of several positive antibodies corresponds to a more favourable prognosis in SLEPAH, with the majority of patients seeing an improvement in their PAH.
